# G6PD deficiency alleles in a malaria-endemic region in the Western Brazilian Amazon

**DOI:** 10.1186/s12936-017-1889-6

**Published:** 2017-06-15

**Authors:** Jamille G. Dombrowski, Rodrigo M. Souza, Jonathan Curry, Laura Hinton, Natercia R. M. Silva, Lynn Grignard, Ligia A. Gonçalves, Ana Rita Gomes, Sabrina Epiphanio, Chris Drakeley, Jim Huggett, Taane G. Clark, Susana Campino, Claudio R. F. Marinho

**Affiliations:** 1 Department of Parasitology, Institute of Biomedical Sciences, University of São Paulo, São Paulo, Brazil; 2 Multidisciplinary Center, Federal University of Acre, Acre, Brazil; 3 LGC Genomics, Hoddesdon, Hertfordshire, UK; 4 Faculty of Infectious and Tropical Diseases, London School of Hygiene and Tropical Medicine, London, UK; 5 Department of Clinical and Toxicological Analyses, School of Pharmaceutical Sciences, University of São Paulo, São Paulo, Brazil; 6 Molecular and Cell Biology, LGC, Teddington, Middlesex UK; 7 School of Biosciences & Medicine, Faculty of Health & Medical Sciences, University of Surrey, Guildford, Surrey UK; 8 Faculty of Epidemiology and Population Health, London School of Hygiene and Tropical Medicine, London, UK

**Keywords:** Malaria, Glucose-6-phosphate dehydrogenase, Genetic variants

## Abstract

**Background:**

*Plasmodium vivax* parasites are the predominant cause of malaria infections in the Brazilian Amazon. Infected individuals are treated with primaquine, which can induce haemolytic anaemia in glucose-6-phosphate dehydrogenase (G6PD)-deficient individuals and may lead to severe and fatal complications. This X-linked disorder is distributed globally and is caused by allelic variants with a geographical distribution that closely reflects populations exposed historically to endemic malaria. In Brazil, few studies have reported the frequency of G6PD deficiency (G6PDd) present in malaria-endemic areas. This is particularly important, as G6PDd screening is not currently performed before primaquine treatment. The aim of this study was to determine the prevalence of G6PDd in the region of Alto do Juruá, in the Western Brazilian Amazon, an area characterized by a high prevalence of *P. vivax* infection.

**Methods:**

Five-hundred and sixteen male volunteers were screened for G6PDd using the fluorescence spot test (Beutler test) and CareStart™ G6PD Biosensor system. Demographic and clinical-epidemiological data were acquired through an individual interview. To assess the genetic basis of G6PDd, 24 SNPs were genotyped using the Kompetitive Allele Specific PCR assay.

**Results:**

Twenty-three (4.5%) individuals were G6PDd. No association was found between G6PDd and the number of malaria cases. An increased risk of reported haemolysis symptoms and blood transfusions was evident among the G6PDd individuals. Twenty-two individuals had the G6PDd A(−) variant and one the G6PD A(+) variant. The Mediterranean variant was not present. Apart from one polymorphism, almost all SNPs were monomorphic or with low frequencies (0–0.04%). No differences were detected among ethnic groups.

**Conclusions:**

The data indicates that ~1/23 males from the Alto do Juruá could be G6PD deficient and at risk of haemolytic anaemia if treated with primaquine. G6PD A(−) is the most frequent deficiency allele in this population. These results concur with reported G6PDd in other regions in Brazil. Routine G6PDd screening to personalize primaquine administration should be considered, particularly as complete treatment of patients with vivax malaria using chloroquine and primaquine, is crucial for malaria elimination.

**Electronic supplementary material:**

The online version of this article (doi:10.1186/s12936-017-1889-6) contains supplementary material, which is available to authorized users.

## Background

Glucose-6-phosphate dehydrogenase (G6PD) is an enzyme present in all cells and is involved in the prevention of cellular oxidative damage. This enzyme has a particularly critical function to control the oxidative damage in red blood cells (RBC) and maintain their integrity. In spite of its vital function, the *G6PD* gene, located on chromosome X, has a high genetic diversity and is highly polymorphic. The majority of mutations do not alter the function of the enzyme, but some can lead to a decrease in the enzymatic activity and cause the G6PD deficiency (G6PDd) phenotype [1, 2].

G6PDd is very common worldwide and is characterized by a range of enzyme activity levels and associated phenotypes [3]. The majority of cases are asymptomatic but with a high risk of developing acute haemolytic anaemia with exposure to associated triggers. These triggers include fava bean consumption, oxidizing medications such as sulfonamides, nitrofurantoin and primaquine (PQ) treatment used to prevent *Plasmodium vivax* clinical relapses [4].

Severe G6PDd symptoms, including chronic or intermittent haemolytic anaemia, are in general associated with rare allelic variants. The geographical distribution of G6PDd globally is variable, but it is particularly prevalent in malaria-endemic countries [5, 6]. The highest frequencies are reported in Africa and in a belt extending from the Mediterranean to Southeast Asia [6]. The frequencies vary from ~2 to 30%, depending on the country and ethnic group. In the Americas, G6PDd is less common, with frequencies ranging from 0 to ~14% [7].

Two genetic variants (G6PD A(−) and G6PD Mediterranean) are among the most frequent and are prevalent in particular populations [3, 6]. The A(−) genetic variant, which results from two non-synonymous mutations (G202A/A376G) is common in individuals of sub-Saharan African origin [3, 6]. This variant is in general characterized by an enzymatic activity higher than 10% of normal levels [8]. The *G6PD* Mediterranean variant (C563T) is predominant in West Asia (from Turkey, Saudi Arabia to India) [3, 6], and causes some of the most severely deficient phenotypes with <1% enzymatic activity [8]. In Southeast Asia, the prevalence of G6PDd varies widely among regions and ethnic groups and several genetic variants are observed.

In Brazil, a predominance of the African variant (A−) has been reported, and few new mutations identified [7]. To date, there are few estimates of the frequency of G6PDd in malaria endemic areas. This is particularly important as *P. vivax* is the main malaria parasite in Brazil and PQ, which triggers acute haemolytic anaemia in G6PDd individuals, is the recommended drug to prevent *P. vivax* clinical relapses. Therefore, to predict the potential incidence of primaquine (PQ)-associated acute haemolytic anaemia and determine the likely need for G6PDd testing, this study aimed to estimate the prevalence of phenotypic G6PDd and identify the main genetic variants in a malaria-endemic area in the Western Brazilian Amazon.

## Methods

### Study population and region

A cross-sectional study was carried out from June to August 2016 in the Amazonian region of the Alto do Juruá valley (Acre, Brazil). This region, which has high risk for malaria transmission, includes 5 main cities: Cruzeiro do Sul (7°37′51″S, 72°40′12″W), Rodrigues Alves (07°44′31″S, 72°38′49″W), Mâncio Lima (07°36′50″S, 72°53′4″W), Marechal Thaumaturgo (08°56′27″S, 72°47′31″W) and Porto Walter (08°16′08″S, 72°44′38″W), with a total population in 2010 of 131,505 inhabitants (66,792 males) [9]. Participant volunteers were recruited by a media (radio, TV) campaign where they were encouraged to visit participating study clinics and schools in the Vale do Juruá region. A total of 653 men living in the Juruá Valley region agreed to participate in the study. After exclusions, a total of 516 male individuals from 18–90 years old were selected for analyses (Additional file 1). Demographic and clinical-epidemiological data such as ethnic origin (Afro-descendants, European descents, Mestizo (combined Brazilian native, with European and African ancestry), previous malaria infection and previous clinical complications, particularly anaemia, black urine and need for blood transfusion, were acquired through an individual interview. The data were recorded in a standard questionnaire applied by trained interviewers.

### Evaluation of G6PD deficiency and malaria infection

Four ml of venous blood were collected from each person using EDTA tubes (BD Vacutainer). The qualitative determination of G6PDd in whole blood was performed by a fluorescence spot test using the method of Beutler [10] and positive individuals were also tested with the CareStart™ G6PD Biosensor system (ACCESSBIO, INC) for a quantitative measurement by digital puncture. The reliability of the G6PD Biosensor was assessed by using a control strip provided prior to each testing. In addition, quantification of haemoglobin was performed using the Hemocue^®^ portable spectrophotometer for all volunteers. The result of the enzymatic activity was calculated by normalizing the value attained in the Carestart test (U/dl), which is linked to the value of the haemoglobin level measured by the Hemocue (g/dl). All tests were performed immediately after blood collection. At the time of recruitment, for the volunteers who presented signs and/or symptoms of malaria, the diagnosis was made by thick blood smears and read by a local microscopist from the endemic surveillance team as recommended by the Brazilian Ministry of Health. Only 14 individuals were positive. The fact these individuals had malaria at the time of recruitment was not an exclusion factor.

### G6PD genotyping

For molecular characterization of G6PDd, total DNA was obtained from whole blood cells through the use of a commercially available extraction kit (QIAmp DNA Mini Kit, Qiagen), following the manufacturer’s instructions. A total of 24 single nucleotide polymorphisms (SNPs) were genotyped using the Kompetitive Allele Specific PCR (KASP) assay (LGC Genomics, UK).

SNP selection was based on polymorphisms associated with G6PDd. This included SNPs identified in studies performed in Brazil and Latin America, as well as reported by the 1000 Genomes project [7, 11–13]. For each putative SNP sequence, a KASP assay was designed, comprising two allele-specific forward primers, and one common reverse primer. Genotyping reactions were performed using the IntelliQube (LGC, Douglas Scientific, USA) to complete all DNA arraying, sample and reagent dispensing, plate sealing and fluorescence detection steps for SNP genotyping. Reactions were performed in Array Tape with 384 reactions wells per array. To each well, 0.8 μL of genomic DNA suspension was arrayed followed by dispensing 0.8 μL 2× KASP reaction mix with the non-contact dispense jet to create total reaction volumes of 1.6 μL. The final reaction volume of 1.6 µL contained 1× KASP Array Tape Master mix (LGC Genomics, UK), 0.07 µL KASP assay mix and between 0.5 and 5 ng genomic DNA. Thermal cycling was performed using a Hydrocycler (LGC Genomics, UK). The following cycling conditions were used: 15 min at 94 °C; 10 touchdown cycles of 20 s at 94 °C, 60 s at either 63–57 °C or 68–62 °C (dropping 0.6 °C per cycle) depending on primer thermodynamic profile; and amplification of 35 cycles of 20 s at 94 °C, 60 s at 57 °C). Following completion of the PCR, each sample was assigned a genotype based on cluster plot analysis of raw data using Kraken software (LGC Genomics, UK).

### Statistical analysis

The statistical analyses were performed using Stata version 14.1 (STATA Corp, College Station, TX, USA). The prevalence of G6PDd and variants was calculated as proportions with 95% CI (CI 95%). Qualitative data were analysed with the Chi square test or the Fisher Exact-test when indicated. To assess the extent to which ethnic origin, previous malaria exposure and previous clinical complications were associated with G6PDd, odds ratios (OR) with CI 95% were estimated by univariate logistic regression analysis. Adjustment for multiple variables was performed by adding the covariates in a set of multiple logistic-regression models. Test for trend was conducted through the Exact test for trend (modified Wilcoxon test). A two-tailed value of P < 0.05 was considered statistically significant.

## Results

A total of 516 male volunteers from the Juruá Region in the state of Acre were screened for G6PD activity using the Beutler test. The majority of the individuals were of combined Mestizo (European-Amerindian-African) descent (74.6%), followed by European (14.1%) and Afro-descendants (10.3%) (Table 1). Twenty-three individuals were identified has G6PDd (4.5%) and no difference was observed between ethnic groups (p = 0.877). For these individuals, the activity of the G6PD enzyme was measured using the CareStart™ G6PD Biosensor, which gives a quantitative measurement of total enzyme activity. The concordance of the two tests was 100%. The G6PD quantitative results were normalized using haemoglobin (Hb) levels, which were normal for all G6PDd individuals (>13 g/dL) (Additional file 2). The average G6PD activity was 4.3 U/gHb (IQR 3.4–4.9 U/gHb) (Additional file 2). The normal G6PD activity values can vary but according to WHO criteria, the mean normal activity is 16.37 U/g Hb, the upper limit of mild/partial deficiency is 9.82 U/g Hb and 1.64 is the upper limit of total deficiency. None of the G6PDd individuals in this study had total deficiency, but all had values lower than the mild/partial deficiency upper limit.

**Table 1:** Social and clinical characteristics of the study population

Characteristics^a^	G6PD normal (N = 493)	G6PD deficient (N = 23)	Total (N = 516)
Age—years	36.4 ± 14.8	37.9 ± 12.3	36.5 ± 14.7
Ethnic group			
European descendant	70 (14.2)	3 (13.0)	73 (14.1)
Afro- descendant	50 (10.1)	3 (13.0)	53 (10.3)
Mestizo^b^	368 (74.7)	17 (74.0)	385 (74.6)
Others	5 (1.0)	0	5 (1.0)
Haemoglobin—g/dl	15.2 ± 1.3	15.0 ± 1.2	15.2 ± 1.3
ABO-Rh			
A+/A−	65 (13.2)	4 (17.4)	69 (13.4)
B+/B−	17 (3.5)	1 (4.4)	18 (3.5)
AB+/AB−	8 (1.6)	0	8 (1.6)
O+/O−	117 (23.7)	5 (21.7)	122 (23.6)
History of malaria^c^			
Malaria episode	415 (84.2)	21 (91.3)	436 (84.5)
Primaquine treatment	352 (71.4)	17 (73.9)	369 (71.5)
Use of bed nets	358 (72.6)	19 (82.6)	377 (73.1)
Use of insecticide-treated bed nets	244 (68.2)	17 (89.5)	261 (69.2)
Indoor residual spraying	291 (59.0)	15 (65.2)	306 (59.3)
Individual protection	152 (30.8)	8 (34.8)	160 (31.0)

^a^Values are presented as mean ± standard deviation or absolute number (percentage); ^b^ combined Brazilian native, European and African ancestry. ^c^ Malaria episodes were reported as the infections presented during all life; For primaquine treatment the use was considered only for *P. vivax* infections; For the use of bed nets it was considered the habit of use and use the night before the interview; Individual protection includes the use of long-sleeved clothing, insect repellent to the body, aerosol and electric insecticides and use of screen on windows

The clinical history of the volunteers was also investigated, particularly to verify if there is a correlation between G6PDd and malaria, haemolysis symptoms and blood transfusions (Table 2). No difference was observed between the G6PDd and normal groups in relation to the number of malaria episodes (Table 2). However, the number of haemolysis symptoms (anaemia OR 4.81, CI 2.03–11.40, *p* value <0.001; jaundice OR 7.13, CI 3.02–16.85, p value <0.001; black urine OR 4.09, CI 1.75–9.56, p = 0.001), need for hospitalizations (OR 5.48, CI 1.27–23.64, p = 0.023) and blood transfusions (OR 3.33, CI 1.17–9.45, p = 0.024) reported in the G6PDd group was higher than in the G6PD normal group (Table 2).

**Table 2:** Previous clinical complications and malaria episodes

	G6PD normal (N = 493)	G6PD deficient (N = 23)	Total (N = 516)	Crude OR [95% CI]	p value	Adjusted OR [95% CI]^a^	p value
Ethnicity					0.877	–	–
European-descendant	70	3	73	−			
Non-European	423	20	443	0.91 [0.26–3.13]			
Previous episodes of malaria (episodes)					0.363	–	–
<3	258	11	269	1.50 [0.62–3.62]			
≥3	156	10	166	–			
Previous clinical complications							
Anemia	68	10	78	4.81 [2.03–11.40]	<0.001	3.24 [1.26–8.27]	0.014
Jaundice	76	13	89	7.13 [3.02–16.85]	<0.001	4.02 [1.53–10.55]	0.005
Black urine	119	13	132	4.09 [1.75–9.56]	0.001	1.77 [0.68–4.62]	0.241
Need of hospitalization	324	21	345	5.48 [1.27–23.64]	0.023	3.77 [0.83–17.16]	0.086
Need of blood transfusion	38	5	43	3.33 [1.17–9.45]	0.024	1.24 [0.38–4.03]	0.723

*OR* odds ratio

^a^Logistic regression model using clinical complications, need of hospitalization and blood transfusion

The G6PDd individuals were genotyped for 24 SNPs in the *G6PD* gene. These include the polymorphisms for the following variants: G6PD A(−), Mediterranean, Seatle, Chatam, Santamaria, Betica-Selma, and other SNPs that were polymorphic in South America, according to the 1000 Genome data [14], and/or previously associated with protection to severe malaria [15–17]. According to the genotypes, 22 out of 23 G6PDd individuals had the *G6PD* A(−) variant (376 A>G and 202 G>A mutations) (Additional file 3). One individual was G6PD A(+), which only includes the 376 A>G mutation. Almost all remaining SNPs (n = 17) were monomorphic for the wild-type allele, including the Mediterranean, Seatle, Chatam, Santamaria, Betica-Selma variants. Three SNPs had a minor allele frequency (MAF) of 0.04 and were in complete linkage disequilibrium with the 202 G>A variant. One allele had a MAF of 0.31, with no differences in allele frequencies between ethnic groups. A similar frequency was reported in the 1000 Genomes project data for South America (MAF = 0.39) [14].

## Discussion

This is the first study to evaluate the prevalence of G6PD deficiency in the malaria-endemic region of Alto do Juruá, Acre state, located in the western Brazilian Amazon. Approximately 95% of the malaria cases reported in Acre are found in the city of Cruzeiro do Sul and the surrounding Alto do Juruá region, where this study was conducted. This region is characterized by a high prevalence of *Plasmodium vivax* over *Plasmodium falciparum* infections with an Annual Parasitic Index above 200 cases per 1000 inhabitants [18]. In this region in 2016, approximately 27,000 cases of *P. vivax* infections, 6240 of *P. falciparum* and 227 cases of co-infections were reported [18]. Individuals that are *P. vivax*-positive receive a recommended treatment of chloroquine and PQ. The evaluation of G6PDd before PQ treatments is not part of the Brazilian National Programme for Malaria Control, therefore there is a risk for PQ to induce acute haemolytic anaemia in G6PDd individuals.

**Fig. 1:**
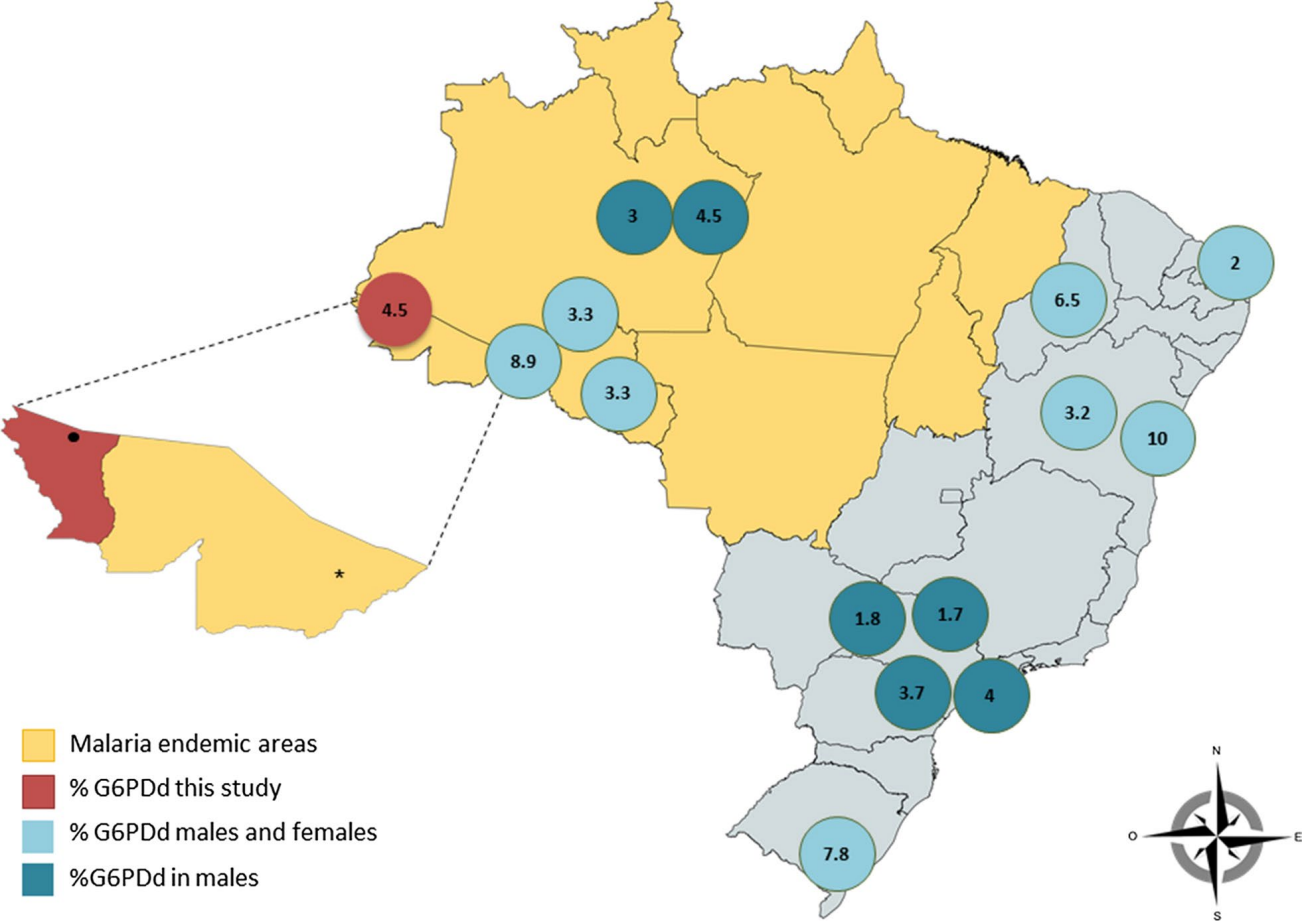
Geographic distribution of G6PD deficiency (G6PDd) frequency (%) reported in Brazil

In this study, the prevalence of G6PDd was evaluated in 516 male volunteers and a frequency of 4.5% was detected, which is similar to those reported elsewhere in Brazil with an overall average of 5.2% (Table 3; Fig. 1). Some regions reported values higher than average (>8%), but study design differences have to be taken into consideration. These include differences in the study population, as in certain cases these involve hospitalized individuals or those with suspected G6PDd. The method used for G6PDd screening should also be taken in consideration.

**Table 3:** Frequency of G6PD genotypes in different regions in Brazil

State	Region	Study location	Study population	N individuals	G6PDd (n)	G6PDd (%)	A− variant	A+ variant		Mediterranean	Seatle	SantaMaria	Others	References
							rs1050828 (G202A)	rs1050829 (A376G)	rs1050828 (G202A)	rs5030868 (C563T)	rs137852318 (G844C)	rs5030872 (A542T, A376G)		
						Males/females	rs1050829 (A376G)							
Acre	Alto do Juruá	Community	Adult males	516	23	4.46	22	1						
Acre	Acrelândia	Home visits	Children (<10 years of age)	1104	98	8.9								Cardoso et al. [26]
Amazonas	Manaus	Community	Ismail Aziz Community	200	6	3.0	5	0	0	0			1	Santana [20]
Amazonas	Manaus	Community	Adult males	1478	66	4.5	56	0	0	10				Santana [21]
Bahia	Salvador	Hospital	Neonates	655	66	8.3/11.6	54	2	2				5	Neto et al. [27]
Pará	Belém	Hospital	G6PD deficient	196	196	NA	161	14	0	0	9	5	5	Hamel [13]
Piauí	Teresina	Community	Adult population	62	4	10/4.8								Ferreira et al. [28]
Rio Grande do Norte	Natal	Hospital	Newborns	400	8	3.9/0	8							Iglessias et al. [29]
Rio Grande do Sul	Porto Alegre	Hospital	Suspected G6PD deficient	348	36	10.3	34							Castro [30]
Rio Grande do Sul	Rio Grande do Sul	Hospital	Newborns	2799	217	8.6/7.0								Castro [31]
Rio Grande do Sul	Porto Alegre	hospital	Newborns (50% presenting jaudice)	490	22	4.5	14			0			8	Carvalho [32]
Rio Grande do Sul	Porto Alegre	Hospital	Patients with jaudice	173	14	8.7								Giovelli et al. [33]
Rondônia	Porto Velho	Diagnostic Center	Malaria suspected cases	122	4	5.8/0								Katsuragawa [34]
São Paulo	Campinas	Hospital	G6PD deficient	150	150	NA	146	0	0	3			1	Saad [37]
São Paulo	Bragança Paulista	Hospital	Adult males-blood donors	4621	80	1.7		Not investigated	79	0				Compri [35]
São Paulo	São Paulo	Community	Adult males	373	15	4.0	12	0	0	0	2		1	Oliveira [12]
São Paulo	Araraquara	Hospital	Adult males-blood donors	5087	89	1.7	86	0	0	3				Ferreira et al. [38]
São Paulo	Bauro	Health Centre	Children males	2657	97	3.7								Nicolielo et al. [36]

Clinical history was explored in this study. Around 82.6% of the G6PDd individuals have had at least one episode of anaemia, jaundice or black urine, compared to 38.5% in G6PD normal individuals. Also, blood transfusions occurred more frequently in G6PDd than normal individuals (21.7 and 7.7% respectively). Malaria episodes were reported in both groups at the same frequency, indicating that probably G6PDd does not protect against *P. vivax* infection. Other studies in Brazil have also reported an absence of association between G6PD phenotypes and the number of previous episodes of malaria (reviewed in [19]). In contrast, some studies found that G6PDd individuals were less likely to report the occurrence of malaria episodes [20, 21], and this could be due to: (i) the small number of subjects with G6PDd included in almost all studies, (ii) studies have relied on patients self-reporting the number of clinical episodes, and (iii) Plasmodium species is not determined and protection could be species-specific. Other factors such as other haemoglobinopathies could also be important confounders. In Brazil, haemoglobin S was detected in 11.1% of the G6PDd individuals, and other regions in South America have reported similar values. In this study, it was not possible to investigate if G6PDd protects against malaria severity as previously reported [15–17, 22].

The prevalence of genetic variants associated with G6PDd were investigated, and 22 out of 23 individuals were detected with the G6PDd A(−) variant, and one individual with the A(+) variant. The prevalence of the G6PDd A(−) variant agrees with data reported in Brazil, where an average of 86% of individuals have the A(−) variant (Table 3). This variant has been detected in a Brazilian fatal primaquine-Induced haemolysis patient with *P. vivax* malaria [23]. Therefore, it is important to recognize that G6PD A(−), may result in a severe and life-threatening haemolysis on intake of PQ. The Mediterranean variant is the second most reported in Brazil [7], but it was not present in this population. Other South American countries have also reported similar frequencies [7, 24].

The Brazilian population is quite heterogeneous, with a high degree of admixture from different ethnic origins. Besides the native Amerindians, Brazilians descend from African populations from diverse regions and migrants from Europe (Portugal, Italy, Spain, Germany) and Asia (Japan). The frequency of G6PDd among ethnic groups was investigated and an increase of G6PDd was found in Afro-descendants (5.6%) but this difference was not significant when compared to European descendants (4.1%) and those of combined Mestizo ancestry (4.4%) (p = 0.918). The data from this and other studies suggest that the Brazilian population is quite homogeneous regarding G6PDd, regardless of the ethnic groups.

This study is one of few that investigated G6PDd in an area endemic for malaria in Brazil. Fewer than one in three studies were performed in malaria-affected areas and focused on Manaus, Acre and Rondônia, with an absence of information available in other malaria-endemic areas. In Brazil, PQ is used in *P. vivax* infected patients to prevent malaria relapse episodes, but in the absence of informative G6PDd screening. The complete treatment of *P. vivax* malaria advised by the Brazilian Ministry of Health includes a 3 day dose of chloroquine (25 mg/kilo of body weight) and a 7 day (0.5 mg/kg) or 14 day (0.25 mg/kg) scheme of PQ treatment. A short 7 day scheme doubling the PQ dose in order to avoid patients low adhesion to the prolonged treatment is also accepted.

In the Juruá Valley region, the data suggest that one in every 23 male individuals could be G6PDd and, therefore, at risk of haemolysis after PQ treatment. In the Brazilian Amazon several reports have associated G6PDd with a considerably higher risk of haemolysis in patients using PQ, and lethal cases have also been reported (reviewed in [19]). G6PDd screening should be considered by the Malaria Control programmes before PQ administration. Moreover, two studies have shown that the use of PQ in G6PDd men represents a heavy burden on the Brazilian public health service, and G6PDd detection would result in better use of public resources [23, 25].

## Conclusion

In this study, the prevalence of G6PDd was evaluated in a malaria-endemic region in the Western Amazon region, and detected a frequency of 4.5%. The G6PDd A(−) variant was responsible for almost all G6PDd (22/23 individuals). Because this region is endemic for *P. vivax*, PQ treatment is routinely used to prevent relapses from the dormant hypnozoites in the liver. Consequently, one in each 23 males could be G6PDd and at risk to develop haemolytic anaemia upon PQ administration. In order to decide the importance of routine G6PDd screening, particularly if complete treatment of patients with vivax malaria is needed to accelerate elimination of the disease, it is important to consider the cost and feasibility of screening and the risk of severe haemolytic response at different levels of G6PDd.

## Additional files

**Additional file 1.** Flow diagram of the study detailing exclusion criteria.

**Additional file 2.** Characteristics of G6PD deficient individuals and G6PDd screening results.

**Additional file 3.** List of single nucleotide polymorphisms analyzed.

## Abbreviations

G6PD: glucose-6-phosphate dehydrogenase
PQ: primaquine
