# Supplementary figures and images for: G6PD deficiency alleles in a malaria-endemic region in the Western Brazilian Amazon
Source: Malar J. 2017 Jun 15;16:253. doi: 10.1186/s12936-017-1889-6 (PMC5471696; doi:10.1186/s12936-017-1889-6)

**Additional file 1. Flow diagram of the study detailing exclusion criteria.**

**
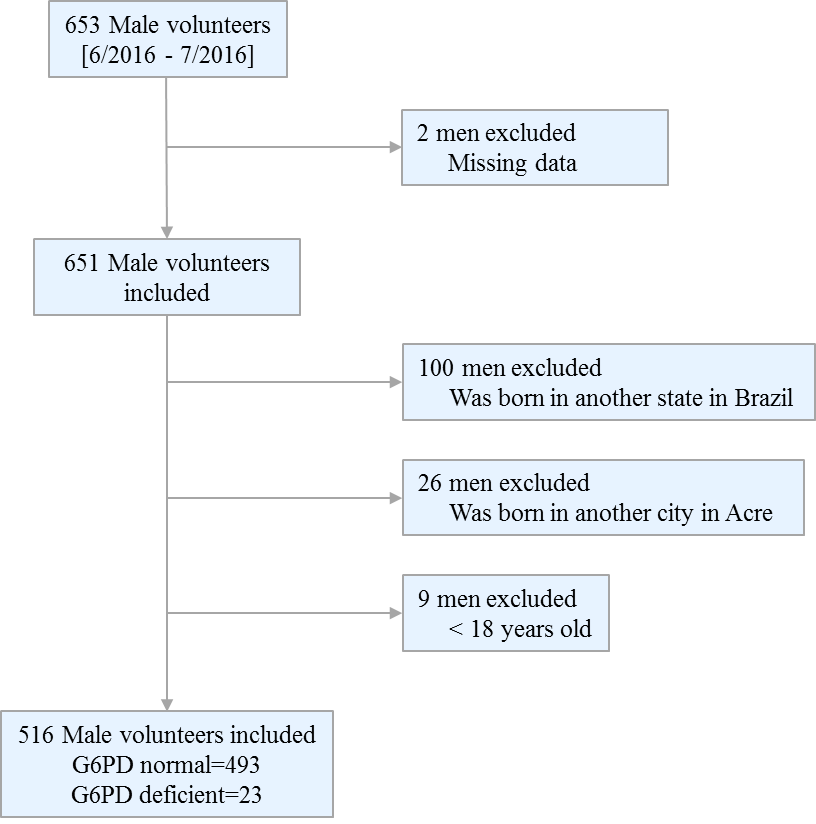
**

Supplement: Supplementary file 1 — Additional file 1. Flow diagram of the study detailing exclusion criteria. [file 12936_2017_1889_MOESM1_ESM.docx]
