# Supplementary material for: G6PD deficiency alleles in a malaria-endemic region in the Western Brazilian Amazon
Source: Malar J. 2017 Jun 15;16:253. doi: 10.1186/s12936-017-1889-6 (PMC5471696; doi:10.1186/s12936-017-1889-6)
Supplement: Supplementary file 2 — Additional file 2. Characteristics of G6PD deficient individuals and G6PDd screening results. [file 12936_2017_1889_MOESM2_ESM.docx]

| **Additional file 2 Characteristics of G6PD deficient individuals and G6PDd screening results.** | | | | | | |
| --- | --- | --- | --- | --- | --- | --- |
| **Ethnic group** | **Age** | **Blood Group*** | **Hb (g/dl)** | **CareStart (U/dl)** | **IU/g Hb** | **G6PD variant** |
| Afro-descendant | 28 | O+ | 14.7 | 38 | 2.6 | A- |
| Afro-descendant | 39 | B- | 13 | 73 | 5.6 | A- |
| Afro-descendant | 45 | NI | 16.7 | 66 | 4.0 | A- |
| Mestizo | 23 | NI | 14.3 | 41 | 2.9 | A- |
| Mestizo | 41 | A- | 17.7 | 56 | 3.2 | A- |
| Mestizo | 29 | NI | 15.1 | 48 | 3.2 | A- |
| Mestizo | 44 | NI | 14 | 48 | 3.4 | A- |
| Mestizo | 23 | NI | 16.9 | 65 | 3.8 | A- |
| Mestizo | 61 | O+ | 15.2 | 59 | 3.9 | A- |
| Mestizo | 43 | NI | 14.5 | 57 | 3.9 | A- |
| Mestizo | 48 | NI | 14.8 | 61 | 4.1 | A- |
| Mestizo | 52 | NI | 13.8 | 64 | 4.6 | A+ |
| Mestizo | 26 | A+ | 15.9 | 77 | 4.8 | A- |
| Mestizo | 47 | O+ | 16.1 | 79 | 4.9 | A- |
| Mestizo | 51 | O+ | 14 | 81 | 5.8 | A- |
| Mestizo | 39 | NI | 14.7 | 110 | 7.5 | A- |
| Mestizo | 21 | NI | 16.3 | . | . | A- |
| Mestizo | 49 | NI | 16.5 | . | . | A- |
| Mestizo | 24 | A+ | 14 | . | . | A- |
| Mestizo | 46 | NI | 14.6 | . | . | A- |
| European-descendant | 18 | NI | 14 | 79 | 5.6 | A- |
| European-descendant | 18 | A+ | 14.5 | . | . | A- |
| European-descendant | 18 | O- | 13.8 | . | . | A- |
| ***NI: No information** |  |  |  |  |  |  |
