# Supplementary material for: G6PD deficiency alleles in a malaria-endemic region in the Western Brazilian Amazon
Source: Malar J. 2017 Jun 15;16:253. doi: 10.1186/s12936-017-1889-6 (PMC5471696; doi:10.1186/s12936-017-1889-6)
Supplement: Supplementary file 3 — Additional file 3. List of single nucleotide polymorphisms analyzed. [file 12936_2017_1889_MOESM3_ESM.docx]

|  |  |  | | |
| --- | --- | --- | --- | --- |
| **G6PD Variant** | **Polymorphism** | **MAF*** |  |  |
| A- | rs1050828 | 0.043 | |  |
|  | rs1050829 | 0 | |  |
| Mediterranean | rs5030868 | 0 | |  |
| Chatam | rs5030869 | 0 |  |  |
| SantaMaria | rs5030872 | 0 |  |  |
| Seattle | rs137852318 | 0 |  |  |
|  | rs76723693 | 0 |  |  |
|  | rs2230037 | 0 |  |  |
|  | rs35228794 | 0.31 |  |  |
|  | rs2230036 | 0 |  |  |
|  | rs1894260 | 0 |  |  |
|  | rs73573478 | 0 |  |  |
|  | rs2515906 | 0 |  |  |
|  | rs762515 | 0 |  |  |
|  | rs111827785 | 0 |  |  |
|  | rs1050757 | 0 |  |  |
|  | rs2515904 | 0.043 |  |  |
|  | rs137852328 | 0 |  |  |
|  | rs2515905 | 0.043 |  |  |
|  | rs762516 | 0.043 |  |  |
|  | rs2071429 | 0 |  |  |
|  | rs113492957 | 0 |  |  |
|  | rs2004651 | 0 |  |  |
|  | rs2230037 | 0 |  |  |

*all 23 individuals were genotyped
